# Having a monk in the family and all-cause mortality: a seven-year prospective cohort study

**DOI:** 10.1017/ehs.2025.1

**Published:** 2025-02-17

**Authors:** Liqiong Zhou, Yuan Chen, Erhao Ge, Aijie Zhang, Yasi Zhang, Juan Du, Ruth Mace, Yiqiang Zhan

**Affiliations:** 1 Department of Epidemiology, School of Public Health (Shenzhen), Sun Yat-Sen University, Shenzhen, China; 2 Unit of Integrative Epidemiology, Institute of Environmental Medicine, Karolinska Institute, Stockholm, Sweden; 3 Department of Anthropology, University College London, London, UK; 4 State Key Laboratory of Grassland Agro-ecosystem, College of Ecology, Lanzhou University, Lanzhou, China; 5 Institute for Advanced Study in Toulouse, Université Toulouse, Toulouse, France

**Keywords:** religious celibate monks, Amdo Tibetans, Cox mixed-effects regression, all-cause mortality

## Abstract

Religious celibate monks at the household level possibly reduce all-cause mortality risk among non-monk older Tibetans. This study aims to investigate the association between having a celibate monk in a family and the all-cause mortality of non-monk household members in a Tibetan population. Baseline interviews were conducted for 713 agropastoral Amdo Tibetans aged ≥50 years residing in the eastern Tibetan Plateau from 2016 to 2017. The Cox mixed-effects regression model was used to estimate the association between having a celibate monk in a household and the mortality risk of other non-monk household members. Potential confounders included age, sex, household size, educational attainment, household wealth (measured as the number of yaks), marital status, and annual expenditure. During a median follow-up of 7 years, 54 deaths were identified. The results showed that people living in households with celibate monks had a lower risk of all-cause mortality (hazard ratio: 0.31, 95% confidence interval: 0.14, 0.67) as compared with those living in households without celibate monks. The results remained robust after controlling for confounders, suggesting that religious celibate monks at the household level were associated with lower all-cause mortality among non-monk older household members.

## Graphical abstract

**Figure:**
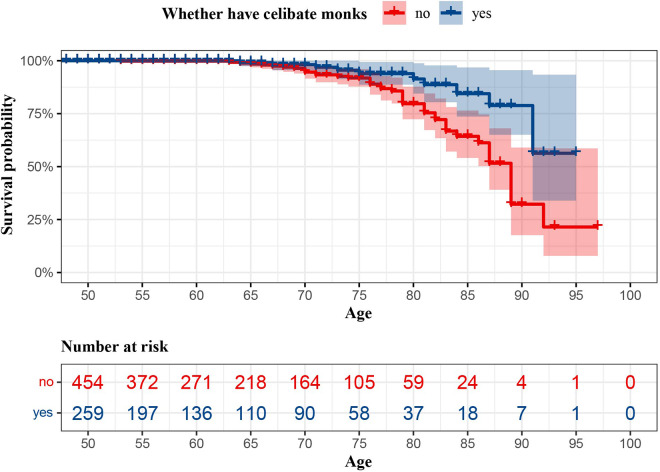
Graphical Abstract:

## 1. Introduction

The associations between religious factors and health have long been of interest in anthropology and has more recently garnered attention from the medical community (Emile, 1973; Koenig, 2008; Koenig, King & Carson, 2012). Religious factors examined in the literature mainly included self-assessments of religiosity and spirituality, regular attendance at religious services (which enhances social support and community connections), and religious celibacy, most of which tended to favour health and longevity (Chen, Zhao & Wang, 2020; Koenig et al., 2012). For example, a study involving 36,613 participants from the Black Women’s Health Study, aged 21–69 years, reported a negative association between religious service attendance and all-cause mortality during the follow-up period from 1995 to 2013 (VanderWeele et al., 2017). Similarly, another study with 8805 respondents aged 80 to 105 from the 1998 and 2000 Chinese Longitudinal Healthy Longevity Surveys (CLHLS) reported that religious activities reduced mortality by 13% (Zhang, 2008). Furthermore, a cohort study conducted on 2987 Danes aged 40 and above, interviewed in SHARE from 2004 to 2007 and followed up until 2018, revealed a 44% reduction in the risk of death among women actively participating in religious services (Ahrenfeldt, Möller, Hvidt, VanderWeele & Stripp, 2023). A meta-analysis of 42 independent samples showed significantly lower mortality of people with high religious engagement (McCullough, Hoyt, Larson, Koenig & Thoresen, 2000). Indeed, some religious groups proscribe or prohibit certain behaviours that are linked to health, for example, bans on the use of tobacco and alcohol (Hassan, Agabani, Ahmed, Shapiro & Le Foll, 2023; King, 2023). A study revealed that the greater frequency of attendance at religious services was associated with lower smoking prevalence (Gillum, 2005). Additionally, many religions advocate respecting and caring for the body through a regular exercise regimen and a more nutritious diet, which can support a healthier lifestyle (McGuire, 2008). Religious practices, such as tolerance, self-control, harmony, inner peace, and wisdom, which may contribute to positive expectations like hope and optimism, and provide spiritual well-being and resources beneficial to longevity, particularly among socially disadvantaged groups like the elderly (Ai, Peterson, Tice, Bolling & Koenig, 2004). Religious practices can boost reputation (CaiRangDongZhi, Du & Mace, 2023; Power, 2017) and hold significant value when considering resources like education, income, and social participation (Schieman, Nguyen & Elliott, 2003).

Religious celibacy is a puzzling institution from the social demographic perspective, as it contradicts the principles of reproductive fitness (Waldner, 2001). Despite this, it remains popular in major religious traditions such as Buddhism, Christianity, Hinduism, Jainism, and Sufi Islam, without a clear explanation as to why religious celibates practice sexual abstinence (or reproductive suppression) (Jansen, 2018; Qirko, 2002). In traditional Tibet, the existence of religious celibate monks, whose daily lives centre around religious beliefs, practices, and devotion, not only allows them to offer spiritual support and moral guidance to their family members, exerting a positive influence on their lives, but also bestows upon the household an elevated social status within the community (Ge, DongZhi & Mace, 2024; Jansen, 2018; Wu, 2013). As monks do not inherit wealth from their parents (Hill, 1999; Zhou et al., 2022), they can engage in prayers for the deceased and receive donations from family members, as well as substantial contributions from devotees during large monastery rituals as another way to ease the burden on families or provide financial support (Goldstein, 2010; Stein, 1972). Even though it has become more frequent in recent years for monks to leave monastic life, those who do so still encounter substantial public disapproval and social exclusion, resulting in these cases being very uncommon (Caple, 2019; Zhou et al., 2022).

Our earlier findings have demonstrated that celibacy can improve inclusive fitness of a monk’s close kin, predominantly by suppressing same-sex sibling competition (Micheletti et al., 2022), similar to how the presence of Catholic priests may cause a resource drain beyond the productivity of the family (Deady, Law Smith, Kent & Dunbar, 2006). As previously mentioned, having a celibate monk in the household can enhance wealth and increase the number of children born to brothers, and it does not appear to be costly to the household as a whole in terms of reproductive success; however, it does not seem to be associated with increased wealth or reproductive success of sisters (Micheletti et al., 2023, 2022; Zhou et al., 2022). However, the decline in the number of Catholic nuns, representing service provision, is a strong predictor of the reduction in fertility differences across Europe (Berman, Iannaccone & Ragusa, 2018). Additionally, in another Amdo Tibetan population, older adults are primarily responsible for nurturing their grandchildren within the village, engaging in household tasks, and participating in basic agricultural activities (Du & Mace 2018; Du, Page & Mace, 2022). Notably, Darwinian fitness benefits can also accrue from caring for elders, even if they are past reproductive age, due to their role as allocarers (such as grandparents) who can assist younger generations in raising offspring (Garay, Számadó, Varga & Szathmáry, 2018).

Nonetheless, it remains an open question as to what additional benefits might accrue to household members of a celibate monk, making this cultural practice potentially more advantageous compared to other mechanisms that reduce household resources competition, such as dispersal or infanticide. Although most of the aforementioned studies examined religion at the individual level, the effects at the household level have been seldom investigated. Although it has been shown that Catholic nuns live longer (Snowdon, 2003), whether there is any longevity benefit to being the sibling or household members of a religious celibate has not been explored. Therefore, we aimed to examine the potential association between having celibate monks in a household and the mortality risks of non-monk members in an Amdo Tibetan population.

## 2. Methods

### 2.1. Study population

Our study area is a county in Gansu Province, China, located in the eastern Tibetan Plateau, inhabited by Amdo Tibetans whose agropastoral lifestyle consists of young individuals tending to yak and Tibetan sheep, while residing in pastures that are situated at altitudes ranging from 3000 to 4500 m above sea level. Their primary means of subsistence is agriculture, yak, and sheep husbandry (Du et al., 2018; Zhou et al., 2022). A considerable proportion of Tibetan Buddhist households send a son, typically between the ages of 7 and 10, to a nearby monastery to become a celibate monk, accounting for about 15% of the local male population; although these monks live in monasteries from pre-teenage years, they maintain ties with their natal household and may return home periodically upon completing religious education (Goldstein & Tsarong, 1985; Micheletti et al., 2022; Wei, 2007; Zhou et al., 2022). In this context, reproductive isolation among celibate monks is maintained through spatial segregation and religious doctrine. Boys are sent to independent monasteries to receive religious instruction, thereby minimizing contact with broader society and restricting reproductive opportunities. Recent policy changes now permit children to pursue monastic life while attending school. Typically, they complete their nine years of compulsory education by the age of 13, after which they reside and study exclusively in the monastery.

A sociodemographic survey was initially self-reported in June 2016, and the remaining villages were surveyed and completed in July 2017, excluding monasteries. It included all those residents in the household and also any monks that were originally from that household that were not resident in monasteries (each monk is generally associated with only one household, typically their natal household). Data from both phases were combined to obtain a complete sample comprising 2256 individuals from 328 households. The follow-up survey was conducted in July 2023 with the assistance of a native interpreter involving all 2256 participants from 328 households, with no loss to follow-up. Furthermore, we restricted the sample to individuals over 50 years old who reported personal and household information at the baseline for 713 individuals from 305 households **(Figure S1).** The study of all-cause mortality focused on natal household members aged 50 and above, as this group has a higher baseline risk of mortality, which allows for the observation of more events (deaths), and their more stable health and lifestyle patterns reduce potential confounding factors.

### 2.2. Data preparation

At baseline, having celibate monks in a household was assessed by determining whether any of its members were celibate monks. If the response was affirmative, it was defined as having a celibate monk at the household level. Mortality data were collected in July 2023. Deaths were identified from reports by next of kin. Survival status was ascertained for all participants, excluding the monks. However, reasons for death were not recorded, and no participants emigrated during the study period. Censoring was defined as the last day of follow-up. The following covariates were considered as potential confounders: age, sex, educational attainment, household size, number of yaks, marital status, and annual expenditure. Educational attainment was categorized as illiteracy, primary school, and middle school; household size was recorded as the total number of people in a household excluding celibate monks; marital status is classified as unmarried, married, and widow/widower; household wealth was assessed as the number of yaks owned in one year, as yaks are a much more significant source of income in our study site, compared to wage labour or crop production; annual expenditure was classified based on total medical and living expenses for a household: low (<10,000 CNY), middle (10,000–49,999 CNY), and high (50,000+ CNY).

### 2.3. Statistical analyses

Characteristics of study participants were summarized by sex. We calculated the mortality risk of agropastoral Amdo Tibetans over the age of 50 years with or without celibate monks in the household and used a log-rank test to examine if the probability of survival curve differed. Additionally, mixed-effects Cox regression models were applied, adjusting for age, sex, educational attainment, household size, number of yaks, marital status, and annual expenditure. We fitted five models of the association between having a celibate monk in a household and the mortality risk of non-monk household members. Considering the left-skewed distribution of age, we squared the age variable in the model. The village was set as a random effect of all models to control for any broader ecological differences between field locations. Model 1 included having celibate monks among the household members. Model 2 adjusted for baseline age and sex, whereas Model 3 further controlled for household size. In Model 4, additional variables including the number of yaks and education level were included. Finally, in the composite Model 5, marital status and annual expenditure were incorporated for a more comprehensive analysis.

We then fitted our model set, which was generated by limited tailored combinations of models according to the conditions specified. Then, all candidate alternative models were eventually compared based on the Akaike Interference Criterion (AIC) value, considering subsets of the provided ‘full model’. Ultimately, the Cox mixed-effects model with the lowest AIC value was selected as our best-fitting model and its model coefficients were presented in a forest plot.

All statistical analyses were performed utilizing R 4.2.2 (R Core Team, 2022). The coxme package was employed to evaluate the proportional hazard model (Therneau, 2022). Hazard ratio (HR) was calculated as the effect measurement for all models, with a 95% confidence interval (CI) provided. *P* < 0.05 was considered to be statistically significant.

## 3. Results

### 3.1. Characteristics of the study participants

Table 1 describes the baseline characteristics of the Tibetans with or without celibate monks in households (*n* = 713), with a mean age of 65.00 ± 12.00 years. During the seven-year follow-up period, 54 deaths were recorded **(Figure S2)**. Among households without celibate monks, there were 43 (9.5%) deaths, whereas households with celibate monks recorded (4.2%) deaths **(Table S1)**. The mean age at death for older adults in households with celibate monks was 78.55 ± 9.30 years, compared to 77.09 ± 8.47 years for those without **(Figure S3)**.

**Table 1.** Demographic characteristics of study population (*n* = 713)

	Overall (*N*=713^1^)	Female (*N*=379^1^)	Male (*N*=334^1^)	*p*-Value^2^ (female vs. ale)
**Age**	65.28 (11.54)	66.12 (11.99)	64.32 (10.95)	0.087
**Household size**	7.63 (2.56)	7.53 (2.55)	7.74 (2.56)	0.4
**Village size**	31 (10)	31 (9)	32 (10)	0.3
**No. yak**	34 (38)	35 (39)	34 (37)	>0.9
**Having celibate monks**	259 (36%)	131 (35%)	128 (38%)	0.3
**Education attainment**				<0.001
Illiteracy	508 (71%)	327 (86%)	181 (54%)	
Primary school	46 (6.5%)	2 (0.5%)	44 (13%)	
Middle school	159 (22%)	50 (13%)	109 (33%)	
**Death**				0.029
No	659 (92%)	358 (94%)	301 (90%)	
Yes	54 (7.6%)	21 (5.5%)	33 (9.9%)	
**Marital status**				<0.001
Unmarried	52 (7.3%)	6 (1.6%)	46 (14%)	
Married	556 (78%)	293 (77%)	263 (79%)	
Widow/widower	105 (15%)	80 (21%)	25 (7.5%)	
**Annual expenditure**				0.9
Low	445 (62%)	235 (62%)	210 (63%)	
Medium	190 (27%)	104 (27%)	86 (26%)	
High	78 (11%)	40 (11%)	38 (11%)	

1 Mean (SD); *n* (%);

2 Chi-squared test.

Table 2 presents the number of monks at the individual and the household levels. At the individual level, there were 666 non-monk household members and 47 celibate monks. At the household level, there were 305 households in total, 202 without celibate monks, and 75 with one monk, 22 with two monks, and 6 with three monks, respectively.

**Table 2.** The number of monks at the individual level and the household level

		Death	
Individual level	Overall (*N* = 713^1^)	No (*N* = 659^1^)	Yes (*N* = 54^1^)
Non-monk	666 (93%)	612 (92%)	54 (8%)
Monk	47 (6.7%)	47 (100%)	0 (0%)
**Household level**	Overall (*N* = 305^1^)	No (*N* = 288^1^)	Yes (*N* = 17^1^)
**Number of monks**			
0	202 (66%)	188 (93%)	14 (7%)
1	75 (25%)	74 (98.6%)	1 (1.4%)
2	22 (7.2%)	20 (90.9%)	2 (9.1%)
3	6 (2.0%)	6 (100%)	0 (0%)

1 *n*(%).

### 3.2. Survival probability

**Figure 1.**
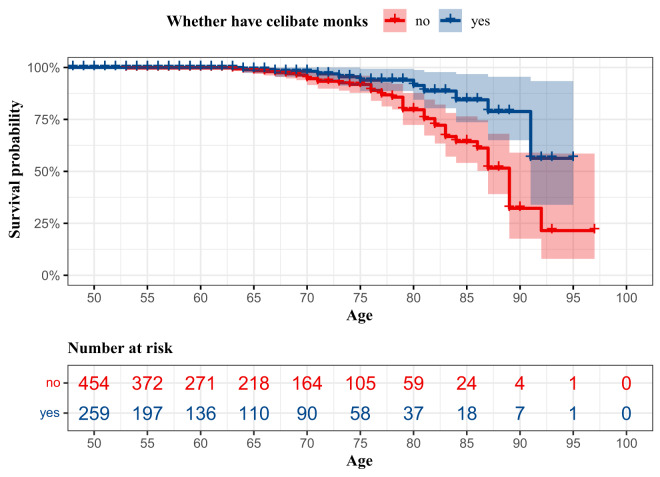
Survival of non-monk older Tibetans who live with and without religious celibate monks.

Figure 1 illustrates the survival probability for non-monk older Tibetans living with and without celibate monks, indicating a statistically significant difference between the two groups (log-rank test: *P* < 0.01). Furthermore, mortality rates among non-monk older Tibetans stratified by sex revealed that men had a lower survival probability than women (log-rank test: *P* < 0.01) **(Figure S4)**.

The sample includes people who died from 2016 to 2023 (*n* = 713, age ≥ 50). The shaded bands represent 95% confidence interval.

### 3.3. Association between having a celibate monk in a household and the all-cause mortality

**Figure 2.**
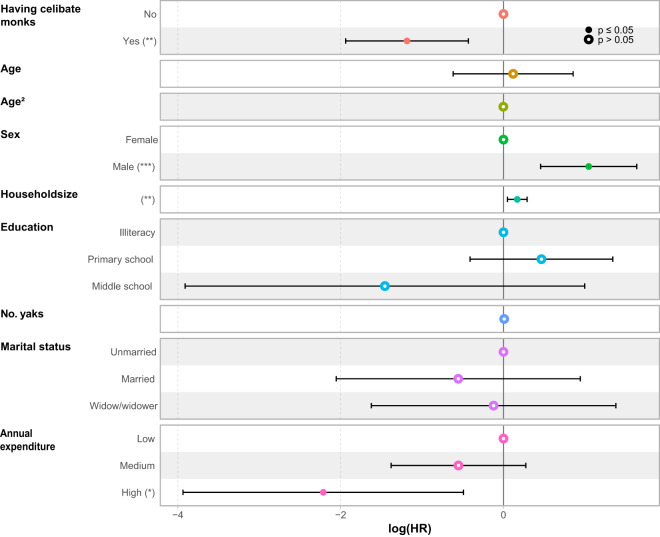
Best models selection of determinants of mortality of non-monk older Tibetans who live with and without celibate monks in households (*n* = 713).

As shown in model 1, having celibate monks in the household was significantly associated with a lower all-cause mortality risk among older non-monk Tibetans. Those who lived with celibate monks in their households had a 64% reduction (HR: 0.36, 95% CI: 0.18, 0.71) in the risk of all-cause mortality as compared with those who were in households without celibate monks (Table 3, Figure 2). In the final model adjusting for age, sex, educational attainment, household size, number of yaks, marital status, and annual expenditure, those with celibate monks in the household had a 69% reduction in the risk of all-cause mortality as compared with those without celibate monks (HR: 0.31, 95% CI: 0.14, 0.67). In sex-specific analyses using Cox mixed-effects models, older non-monk Tibetans with celibate monks in their households had a lower all-cause mortality risk than those without in both male (HR: 0.37, 95% CI: 0.15-0.93) and female (HR: 0.15, 95% CI: 0.03-0.66) groups **(Tables S2 and S3)**.

Statistical significance is indicated in solid circles. Log (HR: Hazard Ratio) above 0 indicates a high risk of death, and log (HR) below 1 indicates a low risk of death.

**Table 3.** Cox mixed-effect regression models for the association between having celibate monk and all-cause mortality (*n* = 713)

	Model 1	Model 2	Model 3	Model 4	Model 5
	HR (95% CI)	HR (95% CI)	HR (95% CI)	HR (95% CI)	HR (95% CI)
**Having celibate monks** (ref: No)					
Yes	**0.36 (0.18–0.71)**	**0.40 (0.20–0.79)**	**0.28 (0.14–0.59)**	**0.27 (0.13–0.58)**	**0.31 (0.14– 0.67)**
**Age**		1.17 (0.66–2.08)	1.24 (0.70–2.19)	1.01 (0.50–2.03)	1.06 (0.50–2.23)
**Age^2^**		1.00 (0.99–1.00)	1.00 (0.99–1.00)	1.00 (0.99–1.00)	1.00 (0.99–1.00)
**Sex** (ref: Female)					
Male		**2.50 (1.43–4.38)**	**2.54 (1.45–4.47)**	**2.56 (1.44–4.53)**	**2.63 (1.48–4.67)**
**Household size**			**1.21 (1.07–1.37)**	**1.21(1.07–1.37)**	**1.18 (1.04–1.34)**
**Education** (ref: Illiteracy)					
Primary school				1.52 (0.63–3.67)	1.49 (0.62–3.58)
Middle school				0.36 (0.04–3.03)	0.30 (0.03–2.86)
**No. yaks**				1.00 (0.99–1.01)	1.01 (1.00–1.02)
**Marital status** (ref: Unmarried)					
Married					0.47 (0.10–2.16)
Widow/widower					0.78 (0.17–3.69)
**Annual expenditure** (ref: Low)					
Medium					0.52 (0.23–1.21)
High					**0.14 (0.02–0.82)**
Random effect	Variance (s.d.)	Variance (s.d.)	Variance (s.d.)	Variance (s.d.)	Variance (s.d.)
Village ID	0.38(0.14)	0.56(0.30)	0.52(0.27)	0.50(0.25)	0.45(0.20)
AIC	489.64	462.02	455.28	458.19	**454.46**

Statistical significance indicated in bold. HR, hazards ratio; CI, confidence intervals. Village ID was set as a random effect (dead = 1, alive = 0, age ≥ 50; person-observations across 11 villages, 2016–2023). HR above 1 indicates a high risk of death and below 1 indicates a low risk of death.

## 4. Discussion

### 4.1. Main findings

This study presented empirical evidence on the potential health implications of having a celibate monk in a household, specifically, older members of their households in a population of agropastoral Amdo Tibetans. Our findings revealed a robust link between the presence of religious celibate monks in the household and lower all-cause mortality risk among older non-monk Tibetans, remaining significant even after adjusting for demographic characteristics. These findings contributed to our understanding of how religion influences health outcomes at household level and provided novel insights into the mechanisms of kin selection for religious celibacy. This insight suggests that the presence of celibate monks, a unique feature of Tibetan Buddhist households, has protective effects on older household members’ mortality risks and have implications for the inclusive fitness interests of a celibate monk’s close kin.

### 4.2. Interpretation of our findings and comparison with the literature

Religious celibate monks at the household level possibly reduce all-cause mortality risk among non-monk older Tibetans through several mechanisms. First, in our case, as celibate monks do not inherit wealth or require resources for marriage and child-rearing from their households (Goldstein, 1978, 2010), having a celibate monk reduces resource competition which enables greater longevity of non-monk members. Previous studies have shown that in this population, men with a monk brother tend to be wealthier and have more children than those with non-celibate brothers (Micheletti et al., 2022; Zhou et al., 2022). It reveals lifelong celibacy could reduce kin competition over household resources. Meanwhile, monks sacrificing their own inheritance and reproductive rights to benefit other members of the household may be adaptive in this ecological and social environment. Alternatively, these costs may lead to monks being seen as true believers or possessing supernatural powers (Singh, 2018; Singh & Henrich, 2020). It is also possible that monks gain great respect in these communities because of their prominent position in society, providing additional material or reputational benefits to their families (Goldstein, 2010). In other societies, individuals who engage in religious activities more frequently have been shown to have higher reputations, which may further benefit other members (CaiRangDongZhi et al., 2023; Ge et al., 2024; Power, 2017).

Additionally, our study found that household size and high annual expenditure were significant predictors of mortality, indicating that these factors could be influencing the observed effects. As our previous work has shown, a monk in the family reduces sibling competition between brothers for household wealth leading to higher wealth and an earlier age at marriage (Micheletti et al., 2022; Zhou et al., 2022). Generally, wealthier and larger families have greater access to medical care, better material conditions, and are more likely to encourage healthy habits, all of which contribute to increased life expectancy over time. This is another mechanism that may further increase the inclusive fitness benefits.

Lastly, having a celibate monk in the household may correlate with a higher likelihood of other household members participating in religious service attendance, which has been associated with lower mortality risks in various populations, including Americans (Case & Deaton, 2015; Ying Chen et al., 2020), Taiwanese (Yeager et al., 2006), Israelis (Litwin, 2007), Chinese (Zhang, 2008), and Danes (Ahrenfeldt et al., 2023). Therefore, the presence of a celibate monk in the family could produce similar protective effects.

What’s more, religious factors and other health outcomes also correlate. For instance, a rural study from the Malawi Religion Project reported a positive relationship between religious activities and mental and physical health among older women (Kendall, 2019). Likewise, levels of religious participation are negatively and significantly correlated with cognitive impairment among the elderly Chinese (Zhang, 2010). Similarly, religious attendance was also associated with maintaining good health behaviours and marital stability for 2676 Alameda County Study participants, spanning ages 17–65 years (Strawbridge, Shema, Cohen & Kaplan, 2001). Among 2812 older individuals aged 65 and over residing in New Haven, Connecticut, participation in public religious activities is associated with improved functional well-being, whereas private religiosity shows a negative correlation with functional impairment and depressive symptoms, although public religious engagement does not impact changes in depressive symptoms (Idler & Kasl, 1992). Religious service attendance has been associated with a reduced risk of suicide among U.S. women (VanderWeele, Li, Tsai & Kawachi, 2016), as evidenced by data from the Nurses’ Health Study II (NHS II) spanning from 2001 through 30 June 2017, and the Health Professionals Follow-Up Study covering the period from 1988 through 31 January 2014 (Chen, Kim & VanderWeele, 2021). Attending religious services was associated with a lower risk of heavy drinking, lower current smoking, fewer depressive symptoms, and higher social well-being (Chen, Cowden, Fulks, Plake & VanderWeele, 2022). However, the results were not always consistent. A cross-sectional study involving individuals aged 60 and above, derived from the 2012 and 2016 Chinese Family Sample Survey, revealed no significant relationship between religiosity and health (Chen et al., 2020). but was generally not associated with subsequent diseases, such as hypertension, stroke, and heart disease in Growing Up Today Study, NHS II and Health and Retirement Study (Chen et al., 2021). Taken together, religious factors encourage better health habits which, in the long run, would lead to increased longevity and decreased morbidity. Although there are few studies that directly examine the mortality of celibate monks and their household members, celibate monks as part of religious factors, and those of existing studies further demonstrate the positive effects of religious factors (religious service attendance, religious beliefs and religious activities, etc.) on physical and mental health. In a household with a celibate monk, household members may be more likely to find spiritual support, which may help reduce stress and improve well-being, thereby slowing mortality among non-monk household members. Religious participation provides psychosocial resources that may compensate for the increased mortality risk associated with adverse socioeconomic conditions in certain vulnerable groups (that is, the elderly). This may another proximate mechanism that helps enhance fitness.

### 4.3. Strengths and limitations

This study possessed several notable strengths. First, the focus of our study is the highly religious modern Tibetan society rather than industrialized society, and by following the participants for up to seven years, the study provided a longer-term observation of the relationship between monks and mortality in their natal household, increasing the reliability and persuasiveness of the findings. Additionally, we concentrate on the influence of celibate monks on death among non-monk family members at the household level, the majority of studies have looked at the relationship between religion and individual mortality for all reasons.

This study also has limitations. First, although our findings regarding the relationship between religion and mortality have become more robust, studies investigating the specific effects of religious celibacy on mortality, the available evidence remains inconclusive and needs to be further explored. Second, as the study focuses on the older agropastoral in Tibetan areas, the generalizability of the results may be limited by cultural differences, and it is difficult to generalize to other social and cultural backgrounds. The initial task was to improve measurement; health data were not measured in the baseline questionnaire, and a large part of the effect of religion on mortality was mediated by health behaviours. Therefore, we added data on religious participation and health during the follow-up. A Cox mixed-effects regression model was used to reduce bias common in observational studies by controlling for potentially confounding variables, introducing random effects allows for more flexibility in dealing with differences between households.

### 4.4. Future research direction

Future research might explore analogous investigations in diverse cultural and social cohorts to augment result extrapolation and perform comparative analyses with various regions or groups. Such endeavours could evaluate the generalizability of findings across different contexts and delve into the ways religion impacts health through psychological and physiological mechanisms. Meanwhile, we should advance the field for longitudinal, cross-national, and health expectancy studies and delve into the specific content of religious and spiritual practices or beliefs, rather than treating them as social or demographic characteristics, such as education or income measures. In addition, it is essential to have longer-term data as well as more comprehensive samples, which future we may be able to overcome. Furthermore, it is unclear to what extent the benefits result from the wealth advantages families gain by sending sons to the monastery where they do not need the resources required for marriage and reproduction, thus reducing sibling competition in their natal household.

In conclusion, we found that the presence of religious celibate monks at the household level was associated with a lower risk of all-cause mortality among older non-monk Tibetans.

## Supporting information

Zhou et al. supplementary material Zhou et al. supplementary material

## Data Availability

The data associated with the sociodemographic analysis are not publicly available in order to preserve participant anonymity, but abridged data are available from the corresponding authors upon reasonable request.

## Supplementary material

The supplementary material for this article can be found at https://doi.org/10.1017/ehs.2025.1.

## Research transparency and reproducibility interest

All procedures involving human subjects were approved by the Research Ethics Committee of the School of Public Health (Shenzhen), Sun Yat-Sen University (No. 2023030). This project secured informed consent at three levels: the Amdo Tibetan Government supervised the research project and given written consent; translator explained data anonymity and given verbal consent from village leaders and research participants. Informed consent was obtained from all participants.
