# Supplementary material for: Having a monk in the family and all-cause mortality: a seven-year prospective cohort study
Source: Evol Hum Sci. 2025 Feb 17;7:e8. doi: 10.1017/ehs.2025.1 (PMC11884931; doi:10.1017/ehs.2025.1)
Supplement: Zhou et al. supplementary material [file S2513843X25000015sup001.docx]

Supplementary Information

| Table S1. Descriptive statistics between religious celibate monk patterns and sociodemographic factors at baseline (n=713) | | | | |
| --- | --- | --- | --- | --- |
|  | | Having celibate monks | |  |
| Characteristic | Overall  (**N** = 713^1^) | No  (**N** = 454^1^) | Yes  (**N** = 259^1^) | p-value^2^ |
| Age | 65.28 (11.54) | 66.12 (11.99) | 64.32 (10.95) | 0.11 |
| Gender |  |  |  | 0.3 |
| Female | 379 (53%) | 248 (55%) | 131 (51%) |  |
| Male | 334 (47%) | 206 (45%) | 128 (49%) |  |
| Household size | 7.63 (2.56) | 7.09 (2.47) | 8.57 (2.43) | <0.001 |
| Village size | 29 (25, 40) | 29 (27, 40) | 31 (25, 33) | 0.4 |
| No. yak | 17 (12, 53) | 12 (10, 40) | 30 (12, 60) | <0.001 |
| Education |  |  |  | 0.5 |
| Illiteracy | 508 (71%) | 323 (71%) | 185 (71%) |  |
| Middle school | 46 (6.5%) | 33 (7.3%) | 13 (5.0%) |  |
| Primary school | 159 (22%) | 98 (22%) | 61 (24%) |  |
| University | 0 (0%) | 0 (0%) | 0 (0%) |  |
| Dead |  |  |  | 0.011 |
| No | 659 (92%) | 411 (91%) | 248 (96%) |  |
| Yes | 54 (7.6%) | 43 (9.5%) | 11 (4.2%) |  |
| Marital status |  |  |  | <0.001 |
| Unmarried | 52 (7.3%) | 16 (3.5%) | 36 (14%) |  |
| Married | 556 (78%) | 368 (81%) | 188 (73%) |  |
| Widow/widower | 105 (15%) | 70 (15%) | 35 (14%) |  |
| Annual expenditure |  |  |  | <0.001 |
| Low | 445 (62%) | 312 (69%) | 133 (51%) |  |
| Medium | 190 (27%) | 109 (24%) | 81 (31%) |  |
| High | 78 (11%) | 33 (7.3%) | 45 (17%) |  |
| ^1^Mean (SD); n (%) | | | | |
| ^2^Wilcoxon rank sum test; Pearson's Chi-squared test; Fisher's exact test | | | | |

Table S2. Parameter estimates in best-fitted candidate models of all-cause mortality in older Amdo Tibetan males (n = 334; dead = 1, alive = 0; age ≥ 50; 2016-2023). Significant effects are in bold. Statistical significance is indicated in bold. HR (Hazard Ratio) above 1 indicates a high risk of death, and below 1 indicates a low risk of death.

|  | Surv mortality male | | |
| --- | --- | --- | --- |
|  | HR | 95%CI | p-value |
| Having celibate monks (ref: No) |  |  |  |
| Yes | 0.37 | 0.15 – 0.93 | 0.035 |
| Age | 1.55 | 0.73 – 3.32 | 0.254 |
| Age^2^ | 1.00 | 0.99 – 1.00 | 0.147 |
| Household size | 1.24 | 1.06 – 1.46 | 0.008 |

Table S3. Parameter estimates in best-fitted candidate models of all-cause mortality in older Amdo Tibetan females (n = 379; dead = 1, alive = 0; age ≥ 50; 2016-2023). Significant effects are in bold. Statistical significance is indicated in bold. HR (Hazard Ratio) above 1 indicates a high risk of death, and below 1 indicates a low risk of death.

|  | Surv mortality female | | |
| --- | --- | --- | --- |
|  | HR | 95%CI | p-value |
| Having celibate monks (ref: No) |  |  |  |
| Yes | 0.15 | 0.03 – 0.66 | 0.012 |
| Age | 0.67 | 0.09 – 5.07 | 0.698 |
| Age^2^ | 1.00 | 0.99 – 1.01 | 0.800 |
| Household size | 1.07 | 0.86 – 1.33 | 0.540 |
| Yak | 1.02 | 1.00 – 1.04 | 0.015 |
| Annual expenditure (ref: Low) |  |  |  |
| Medium | 0.33 | 0.07 – 1.55 | 0.161 |
| High | 0.00 | 0.00 – Inf | 1.000 |


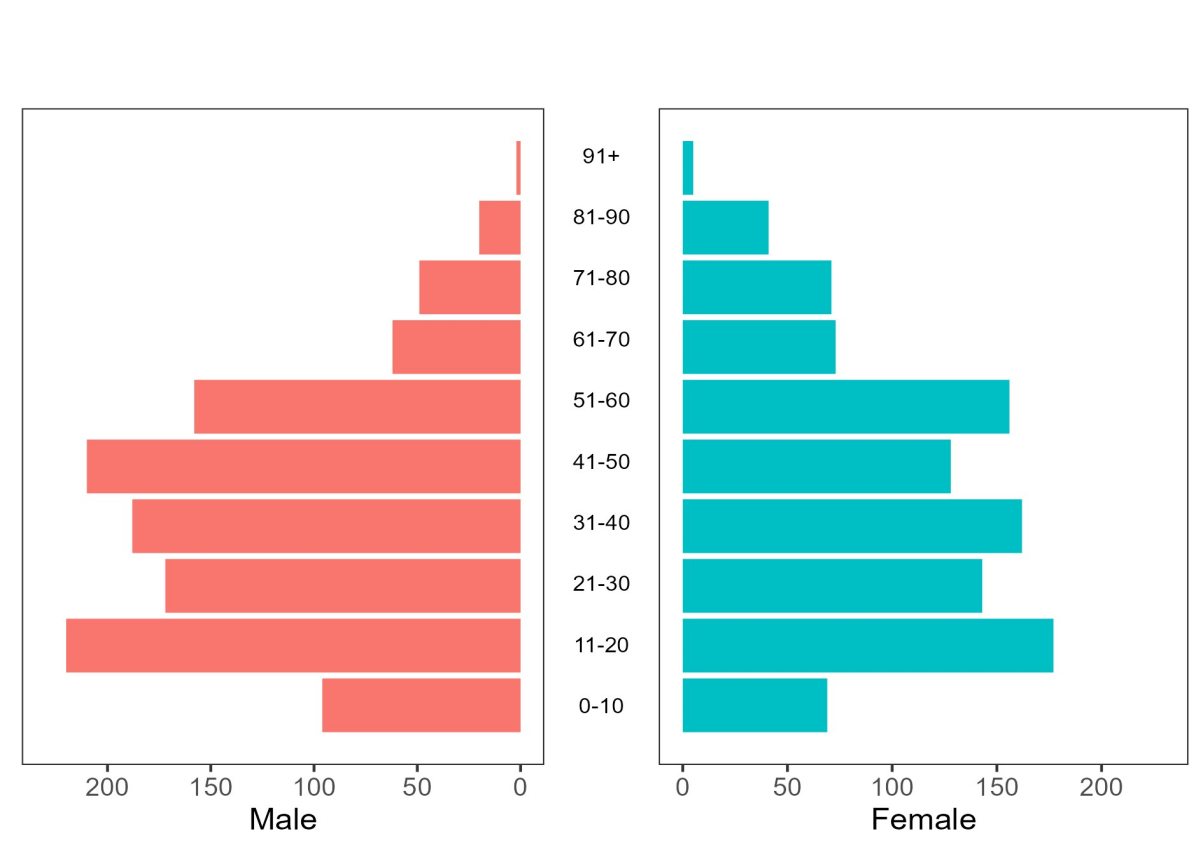
**FiguFigure S1.** Population pyramid at the baseline (n=2256). Each bar represents a 10-year age cohort (0-10, 11-20… through to 91+) and color indicates sex, red is male, blue is female.


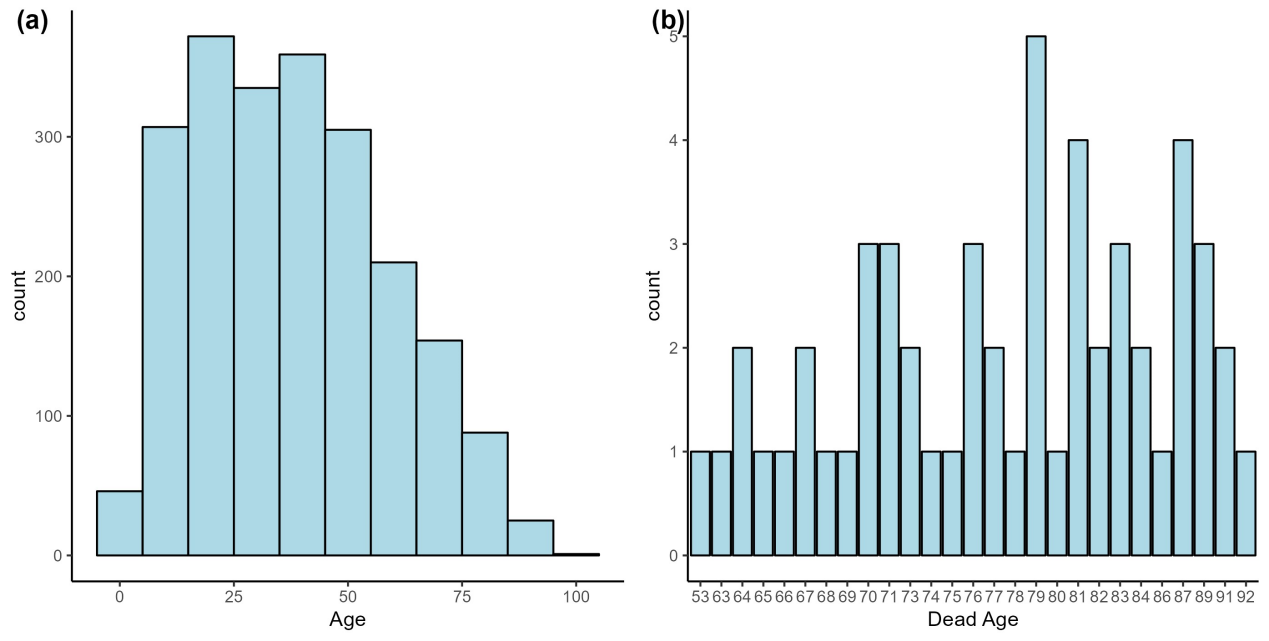
**Figure S2.** (a)Age distribution at the baseline (n=2256); (b) Distribution of deaths by age(n=54)


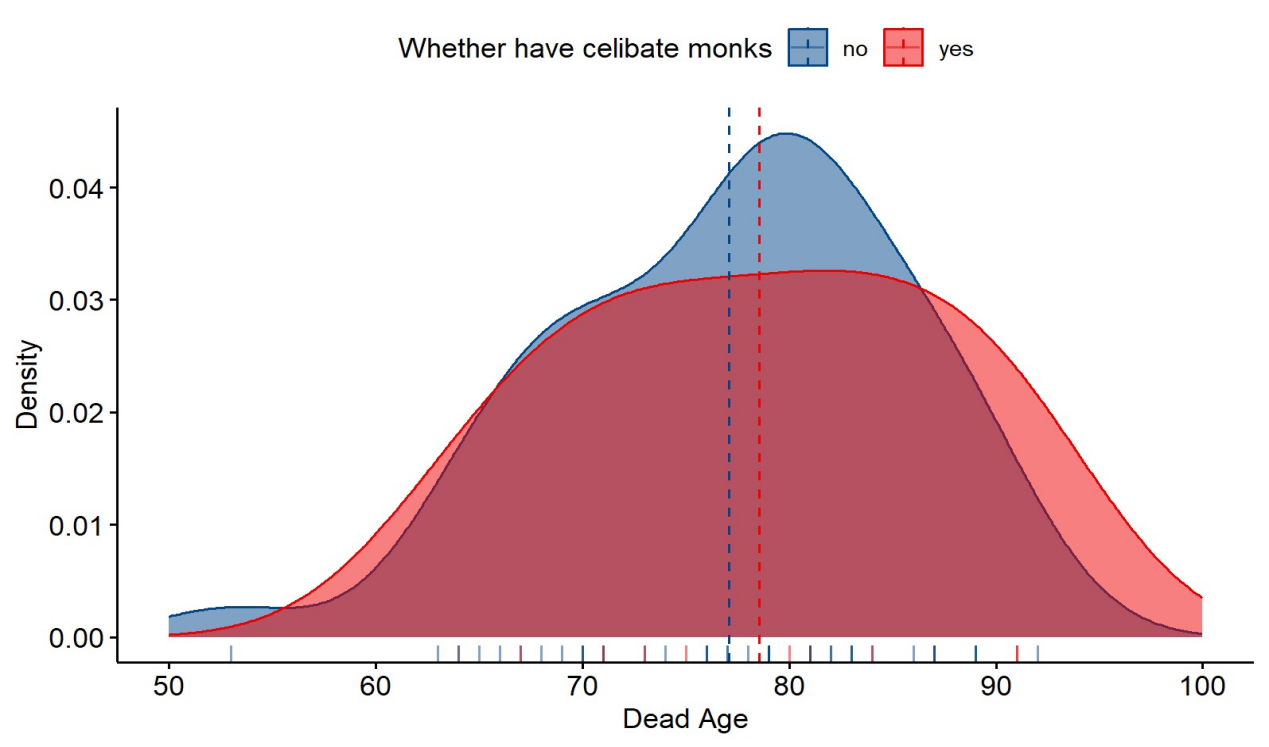
Figure S3. Age-density distribution of elderly deaths by whether a religious celibate monk exists in the household (n=54).


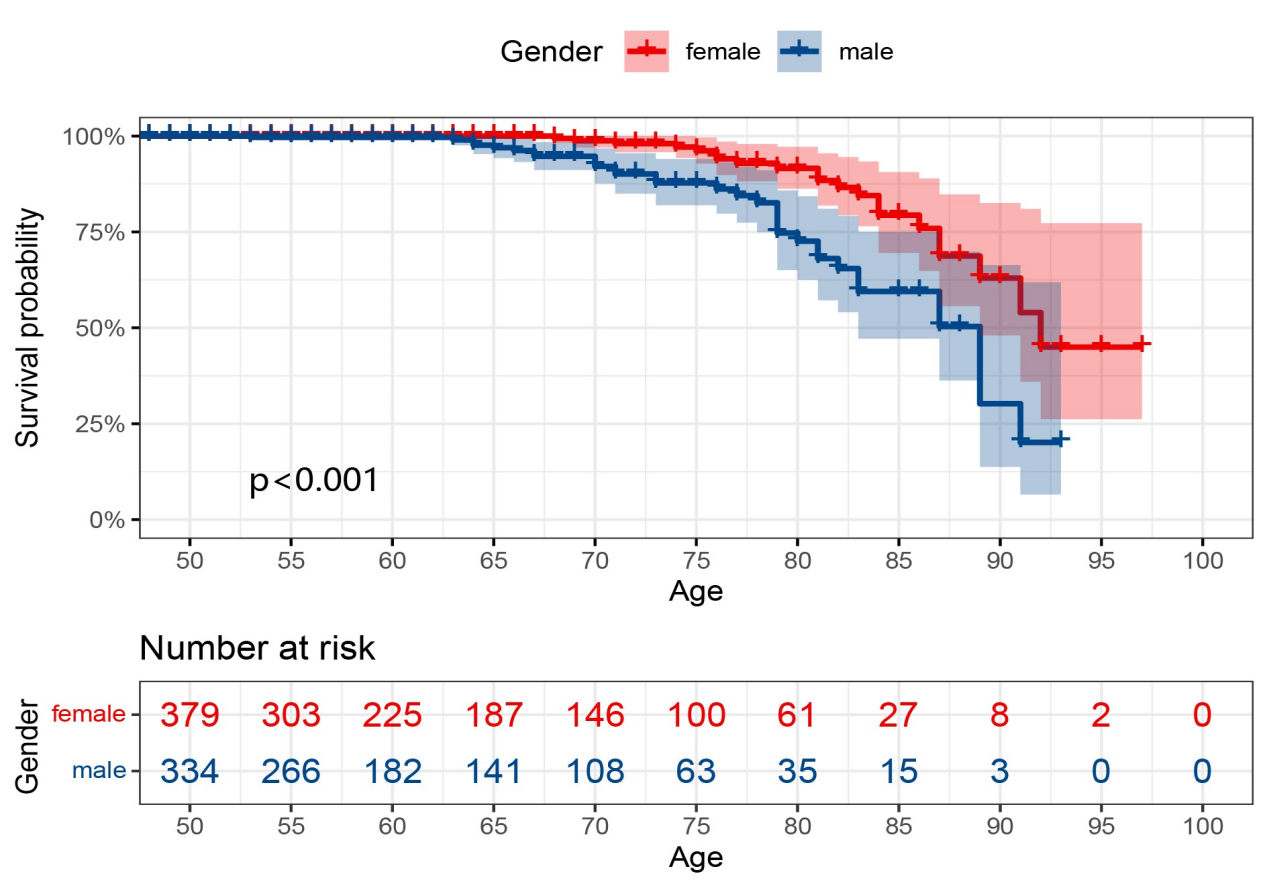


Figure S4. Survival of older adults by sex. The sample includes people who died from 2016 to 2023 (n = 713, age≥50). The shaded bands represent 95% confidence interval
